# Bis(2-trifluoro­methyl-1*H*-benzimidazol-3-ium) naphthalene-1,5-disulfonate

**DOI:** 10.1107/S1600536812000049

**Published:** 2012-01-11

**Authors:** Ming-Liang Liu

**Affiliations:** a Ordered Matter Science Research Center, Southeast University, Nanjing 211189, People’s Republic of China

## Abstract

The asymmetric unit of the title compound, C_8_H_6_F_3_N_2_^+^·0.5C_10_H_6_O_6_S_2_^2−^, consists of one 2-trifluoro­methyl-1*H*-benz­imidazol-3-ium cation and a half naphthalene-1,5-disulfate anion, which are linked by an N—H⋯O hydrogen bond. The anion sits across a centre of symmetry. The atoms of the benzimidazole ring are nearly coplanar (r.m.s. deviation of the fitted atoms = 0.0085 Å) and the triflouromethyl group lies out of this plane. In the crystal, the cations are linked to adjacent anions by N—H⋯O hydrogen bonds, forming a ladder structure parallel to the *a* axis in which the anions form the rungs. Adjacent ladders are linked by weak C—H⋯O inter­actions, forming sheets parallel to the *ac* plane.

## Related literature

The title compound was studied as part of a search for ferroelectric complexes. For background to ferroelectric complexes, see: Fu *et al.* (2011 ▶); Zhang *et al.* (2010 ▶). For related structures, see: Liu (2011*a* ▶,*b* ▶). For graph-set analysis, see: Bernstein *et al.* (1995 ▶).
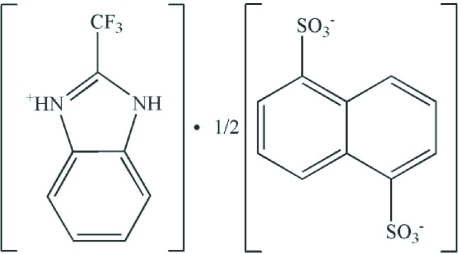


## Experimental

### 

#### Crystal data

2C_8_H_6_F_3_N_2_^+^·C_10_H_6_O_6_S_2_^2−^
*M* *_r_* = 660.56
Triclinic, 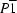

*a* = 9.3910 (19) Å
*b* = 9.4943 (19) Å
*c* = 9.976 (2) Å
α = 109.32 (3)°
β = 96.86 (3)°
γ = 119.59 (3)°
*V* = 685.2 (5) Å^3^
*Z* = 1
Mo *K*α radiation
μ = 0.29 mm^−1^
*T* = 293 K
0.36 × 0.32 × 0.28 mm

#### Data collection

Rigaku Mercury2 diffractometer
Absorption correction: multi-scan (*CrystalClear*; Rigaku, 2005 ▶) *T*_min_ = 0.903, *T*_max_ = 0.921
7215 measured reflections
3136 independent reflections
2361 reflections with *I* > 2σ(*I*)
*R*_int_ = 0.042

#### Refinement

*R*[*F* ^2^ > 2σ(*F* ^2^)] = 0.053
*wR*(*F* ^2^) = 0.132
*S* = 1.10
3136 reflections
227 parameters
36 restraints
H-atom parameters constrained
Δρ_max_ = 0.23 e Å^−3^
Δρ_min_ = −0.42 e Å^−3^

### 

Data collection: *CrystalClear* (Rigaku, 2005 ▶); cell refinement: *CrystalClear*; data reduction: *CrystalClear*; program(s) used to solve structure: *SHELXS97* (Sheldrick, 2008 ▶); program(s) used to refine structure: *SHELXL97* (Sheldrick, 2008 ▶); molecular graphics: *PLATON* (Spek, 2009) ▶; software used to prepare material for publication: *SHELXTL* (Sheldrick, 2008 ▶).

## Supplementary Material

Crystal structure: contains datablock(s) I, global. DOI: 10.1107/S1600536812000049/go2042sup1.cif

Structure factors: contains datablock(s) I. DOI: 10.1107/S1600536812000049/go2042Isup2.hkl

Supplementary material file. DOI: 10.1107/S1600536812000049/go2042Isup3.cml

Additional supplementary materials:  crystallographic information; 3D view; checkCIF report

## Figures and Tables

**Table 1:** Hydrogen-bond geometry (Å, °)

*D*—H⋯*A*	*D*—H	H⋯*A*	*D*⋯*A*	*D*—H⋯*A*
N3—H3⋯O1	0.86	1.81	2.661 (3)	172
N1—H1⋯O2^i^	0.86	1.84	2.650 (3)	155
C12—H12⋯O3^ii^	0.93	2.55	3.440 (3)	159

Symmetry codes: (i) 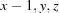; (ii) 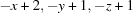.

**Fig. 1.**
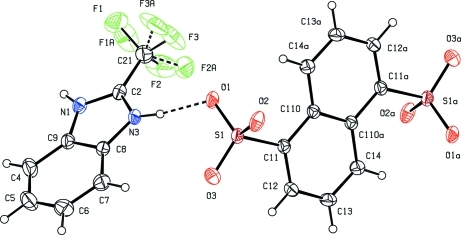
The molecular structure of the title compound, showing the atomic numbering scheme with 30% probability displacement ellipsoids. Atoms with labels suffixed with an a are in a centrosymmetrically related part of the anion.

**Fig. 2.**
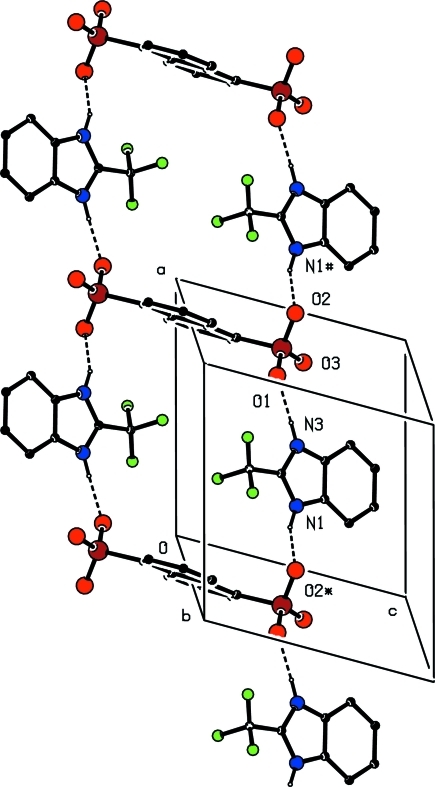
View of the ladder structure which runs parallel to the a axis. For the sake of clarity only the major component of the disordered trifluoromethyl group is shown and H atoms not involved in the hydrogen bonding motif are omitted. Atoms labelled with a * (asterisk) are in the asymmetric unit at (-1+x,y,z) those labelled with a # (hash) are at (1+x,y,z).

## supplementary crystallographic information

### Comment

Recently much attention has been devoted to crystals containing organic ions and inorganic ions due to the possibility of tuning their special structural features and their potential ferroelectrics properties (Fu *et al.*, 2011; Zhang *et al.*, 2010.). In our laboratory, the title compound has been synthesized and its crystal structure is herein reported.

(C_8_H_6_F_3_N_2_)^+^.0.5(C_10_H_6_O_6_S_2_)^2-^ has an asymmetric unit that consists of one 2-trifluoromethyl-1H-benzimidazol cation and a half 1,5-naphthalene disulphate anion linked by a N—H···O hydrogen bond (Fig 1). The atoms of the benzimidazole ring (including H atoms) are nearly coplanar (r.m.s. deviation of the fitted atoms = 0.0085 Å) and the triflouromethyl group which is disordered lies out of this plane. In the crystal structure, the 2-trifluoromethyl-1H-benzimidazole cations are linked the adjacent 1,5-naphthalene disulphate anions by the N1—H1A···O2 and N2—H2a···O1(-1+x,y,z) to form R^4^_4_(26) rings Bernstein *et al.* (1995). These rings are linked to form a ladder structure which runs parallel to the *a* axis. Adjacent ladders are linked by a weak C12—H12···O3(2-x,1-y,1-z) interaction to form sheets which lie parallel to the *ac* plane. The supramolecular structure is further reinforced by a π···π interaction involving the phenyl ring of the benzimidazole cations at (x,y,z) and (1-x,1-y,y,1-z). The centroid to centroid distance is 3.758 (2)Å, the ring perpendicular distance is 3.5120 (14)Å and the offset is 1.336Å.

### Experimental

0.144 g (1 mmol) of 2-trifluoromethyl-1*H*-benzimidazol was firstly dissolved in 30 ml of ethanol, to which 0.288 g (1 mmol) of 1,5-naphthalene disulfonic acid was added to give a solution at the ambient temperature. Single crystals suitable for X-ray structure analysis were obtained by the slow evaporation of the above solution after 3 days in air.

The dielectric constant of the compound as a function of temperature indicates that the permittivity is basically temperature-independent (*ε* = C/(T–T_0_)), suggesting that this compound is not ferroelectric or there may be no distinct phase transition occurring within the measured temperature (below the melting point).

### Refinement

H atoms were placed in calculated positions (N—H = 0.89 Å; C—H = 0.93Å for C*sp^2^* atoms and C—H = 0.96Å and 0.97Å for C*sp^3^* atoms), assigned fixed *U*_iso_ values [*U*_iso_ = 1.2*U*eq(C*sp^2^*) and 1.5*U*eq(C*sp^3^*,*N*)] and allowed to ride. The trifluoromethyl group is disordered over two sites. The site occupancies were refined and restraints were applied to the thermal parameters.

### Crystal data

**Table:**

2C_8_H_6_F_3_N_2_^+^·C_10_H_6_O_6_S_2_^2^^−^	*V* = 685.2 (5) Å^3^
*M**_r_* = 660.56	*Z* = 1
Triclinic, *P*1	*F*(000) = 336
Hall symbol: -P 1	*D*_x_ = 1.601 Mg m^−^^3^
*a* = 9.3910 (19) Å	Mo *K*α radiation, λ = 0.71073 Å
*b* = 9.4943 (19) Å	θ = 3.4–26°
*c* = 9.976 (2) Å	µ = 0.29 mm^−^^1^
α = 109.32 (3)°	*T* = 293 K
β = 96.86 (3)°	Block, colourless
γ = 119.59 (3)°	0.36 × 0.32 × 0.28 mm

### Data collection

**Table:**

Rigaku Mercury2 diffractometer	3136 independent reflections
Radiation source: fine-focus sealed tube	2361 reflections with *I* > 2σ(*I*)
graphite	*R*_int_ = 0.042
CCD_Profile_fitting scans	θ_max_ = 27.5°, θ_min_ = 3.2°
Absorption correction: multi-scan (*CrystalClear*; Rigaku, 2005)	*h* = −12→12
*T*_min_ = 0.903, *T*_max_ = 0.921	*k* = −12→12
7215 measured reflections	*l* = −12→12

### Refinement

**Table:**

Refinement on *F*^2^	Primary atom site location: structure-invariant direct methods
Least-squares matrix: full	Secondary atom site location: difference Fourier map
*R*[*F*^2^ > 2σ(*F*^2^)] = 0.053	Hydrogen site location: inferred from neighbouring sites
*wR*(*F*^2^) = 0.132	H-atom parameters constrained
*S* = 1.10	*w* = 1/[σ^2^(*F*_o_^2^) + (0.0517*P*)^2^ + 0.2409*P*] where *P* = (*F*_o_^2^ + 2*F*_c_^2^)/3
3136 reflections	(Δ/σ)_max_ = 0.011
227 parameters	Δρ_max_ = 0.23 e Å^−^^3^
36 restraints	Δρ_min_ = −0.42 e Å^−^^3^

### Special details

**Table:**

Geometry. All esds (except the esd in the dihedral angle between two l.s. planes) are estimated using the full covariance matrix. The cell esds are taken into account individually in the estimation of esds in distances, angles and torsion angles; correlations between esds in cell parameters are only used when they are defined by crystal symmetry. An approximate (isotropic) treatment of cell esds is used for estimating esds involving l.s. planes.
Refinement. Refinement of F^2^ against ALL reflections. The weighted R-factor wR and goodness of fit S are based on F^2^, conventional R-factors R are based on F, with F set to zero for negative F^2^. The threshold expression of F^2^ > 2sigma(F^2^) is used only for calculating R-factors(gt) etc. and is not relevant to the choice of reflections for refinement. R-factors based on F^2^ are statistically about twice as large as those based on F, and R- factors based on ALL data will be even larger.

### Fractional atomic coordinates and isotropic or equivalent isotropic displacement parameters (Å^2^)

**Table:**

	*x*	*y*	*z*	*U*_iso_*/*U*_eq_	Occ. (<1)
S1	1.05784 (8)	0.72945 (9)	0.37188 (7)	0.03242 (19)	
O1	0.9734 (2)	0.8147 (3)	0.3414 (2)	0.0398 (4)	
O2	1.2456 (2)	0.8519 (2)	0.4164 (2)	0.0440 (5)	
O3	1.0035 (3)	0.6558 (3)	0.4750 (2)	0.0532 (5)	
C11	0.9978 (3)	0.5476 (3)	0.1973 (3)	0.0300 (5)	
C12	0.9359 (3)	0.3826 (3)	0.1952 (3)	0.0362 (6)	
H12	0.9236	0.3679	0.2817	0.043*	
C13	0.8913 (4)	0.2364 (3)	0.0616 (3)	0.0413 (6)	
H13	0.8468	0.1234	0.0594	0.050*	
C14	0.9117 (3)	0.2556 (3)	−0.0659 (3)	0.0365 (6)	
H14	0.8816	0.1559	−0.1531	0.044*	
C110	1.0219 (3)	0.5750 (3)	0.0670 (3)	0.0286 (5)	
N1	0.4697 (3)	0.7605 (3)	0.4315 (2)	0.0360 (5)	
H1	0.3796	0.7607	0.4033	0.043*	
N3	0.7062 (3)	0.7706 (3)	0.4334 (2)	0.0339 (5)	
H3	0.7924	0.7781	0.4066	0.041*	
C2	0.5853 (3)	0.7790 (3)	0.3630 (3)	0.0352 (6)	
C4	0.4442 (4)	0.7162 (4)	0.6660 (4)	0.0518 (7)	
H4	0.3417	0.7094	0.6641	0.062*	
C5	0.5303 (5)	0.7028 (5)	0.7769 (4)	0.0608 (9)	
H5	0.4851	0.6872	0.8528	0.073*	
C6	0.6826 (5)	0.7115 (5)	0.7797 (4)	0.0581 (8)	
H6	0.7369	0.7027	0.8579	0.070*	
C7	0.7555 (4)	0.7326 (4)	0.6704 (3)	0.0468 (7)	
H7	0.8567	0.7366	0.6718	0.056*	
C8	0.6707 (3)	0.7477 (3)	0.5582 (3)	0.0335 (5)	
C9	0.5194 (3)	0.7408 (3)	0.5562 (3)	0.0353 (6)	
C21	0.5768 (4)	0.8018 (5)	0.2213 (4)	0.0523 (7)	
F1	0.5176 (12)	0.9023 (10)	0.2234 (8)	0.0857 (19)	0.653 (12)
F2	0.4588 (17)	0.6460 (7)	0.1027 (5)	0.119 (3)	0.653 (12)
F3	0.7210 (9)	0.8792 (17)	0.2048 (11)	0.113 (3)	0.653 (12)
F1A	0.450 (2)	0.797 (4)	0.1674 (19)	0.114 (5)	0.347 (12)
F2A	0.590 (3)	0.6822 (19)	0.1208 (11)	0.086 (3)	0.347 (12)
F3A	0.720 (2)	0.9515 (16)	0.2418 (15)	0.118 (6)	0.347 (12)

### Atomic displacement parameters (Å^2^)

**Table:**

	*U*^11^	*U*^22^	*U*^33^	*U*^12^	*U*^13^	*U*^23^
S1	0.0354 (3)	0.0412 (4)	0.0305 (3)	0.0274 (3)	0.0148 (3)	0.0162 (3)
O1	0.0412 (10)	0.0540 (11)	0.0460 (10)	0.0373 (9)	0.0240 (8)	0.0255 (9)
O2	0.0311 (10)	0.0428 (11)	0.0480 (11)	0.0247 (9)	0.0066 (8)	0.0066 (9)
O3	0.0827 (15)	0.0612 (13)	0.0382 (11)	0.0485 (12)	0.0333 (11)	0.0294 (10)
C11	0.0283 (12)	0.0354 (13)	0.0308 (12)	0.0195 (11)	0.0136 (10)	0.0162 (10)
C12	0.0424 (14)	0.0436 (15)	0.0357 (13)	0.0274 (13)	0.0205 (11)	0.0245 (12)
C13	0.0527 (17)	0.0303 (13)	0.0433 (15)	0.0215 (13)	0.0205 (13)	0.0214 (12)
C14	0.0407 (14)	0.0325 (13)	0.0369 (14)	0.0204 (12)	0.0153 (11)	0.0164 (11)
C110	0.0250 (11)	0.0332 (13)	0.0311 (12)	0.0174 (10)	0.0121 (9)	0.0162 (10)
N1	0.0281 (10)	0.0427 (12)	0.0436 (12)	0.0242 (10)	0.0138 (9)	0.0189 (10)
N3	0.0331 (11)	0.0426 (12)	0.0399 (11)	0.0274 (10)	0.0193 (9)	0.0212 (10)
C2	0.0352 (13)	0.0353 (14)	0.0383 (14)	0.0233 (12)	0.0143 (11)	0.0143 (11)
C4	0.0491 (17)	0.0541 (18)	0.0566 (18)	0.0284 (15)	0.0328 (15)	0.0261 (15)
C5	0.076 (2)	0.064 (2)	0.0495 (18)	0.0369 (19)	0.0368 (17)	0.0315 (17)
C6	0.075 (2)	0.067 (2)	0.0467 (17)	0.0448 (19)	0.0222 (16)	0.0333 (16)
C7	0.0507 (17)	0.0555 (18)	0.0481 (16)	0.0368 (15)	0.0179 (14)	0.0270 (14)
C8	0.0351 (13)	0.0331 (13)	0.0366 (13)	0.0214 (11)	0.0159 (11)	0.0154 (11)
C9	0.0336 (13)	0.0346 (13)	0.0404 (14)	0.0205 (11)	0.0171 (11)	0.0164 (11)
C21	0.059 (2)	0.069 (2)	0.0493 (18)	0.0446 (18)	0.0237 (16)	0.0337 (17)
F1	0.130 (6)	0.112 (4)	0.072 (4)	0.096 (4)	0.034 (3)	0.056 (3)
F2	0.187 (8)	0.079 (3)	0.037 (2)	0.052 (4)	0.009 (3)	0.0200 (19)
F3	0.086 (4)	0.235 (9)	0.130 (6)	0.112 (5)	0.079 (4)	0.151 (6)
F1A	0.083 (7)	0.238 (15)	0.095 (9)	0.116 (9)	0.044 (6)	0.108 (10)
F2A	0.161 (10)	0.099 (7)	0.036 (4)	0.096 (7)	0.043 (5)	0.031 (4)
F3A	0.160 (11)	0.066 (5)	0.068 (5)	0.016 (5)	0.056 (6)	0.041 (4)

### Geometric parameters (Å, °)

**Table:**

S1—O3	1.435 (2)	N3—H3	0.8600
S1—O2	1.451 (2)	C2—C21	1.498 (4)
S1—O1	1.4547 (18)	C4—C5	1.369 (5)
S1—C11	1.774 (3)	C4—C9	1.395 (4)
C11—C12	1.367 (3)	C4—H4	0.9300
C11—C110	1.430 (3)	C5—C6	1.388 (5)
C12—C13	1.395 (4)	C5—H5	0.9300
C12—H12	0.9300	C6—C7	1.373 (4)
C13—C14	1.366 (3)	C6—H6	0.9300
C13—H13	0.9300	C7—C8	1.384 (4)
C14—C110^i^	1.414 (3)	C7—H7	0.9300
C14—H14	0.9300	C8—C9	1.386 (3)
C110—C14^i^	1.414 (3)	C21—F1A	1.223 (11)
C110—C110^i^	1.419 (5)	C21—F3	1.246 (7)
N1—C2	1.321 (3)	C21—F2A	1.311 (8)
N1—C9	1.380 (3)	C21—F2	1.314 (6)
N1—H1	0.8600	C21—F3A	1.314 (12)
N3—C2	1.312 (3)	C21—F1	1.317 (6)
N3—C8	1.384 (3)		
			
O3—S1—O2	113.71 (13)	N1—C2—C21	124.3 (2)
O3—S1—O1	112.87 (11)	C5—C4—C9	116.0 (3)
O2—S1—O1	110.44 (11)	C5—C4—H4	122.0
O3—S1—C11	107.83 (12)	C9—C4—H4	122.0
O2—S1—C11	104.13 (11)	C4—C5—C6	122.2 (3)
O1—S1—C11	107.24 (11)	C4—C5—H5	118.9
C12—C11—C110	121.5 (2)	C6—C5—H5	118.9
C12—C11—S1	117.00 (18)	C7—C6—C5	121.9 (3)
C110—C11—S1	121.37 (17)	C7—C6—H6	119.0
C11—C12—C13	119.2 (2)	C5—C6—H6	119.0
C11—C12—H12	120.4	C6—C7—C8	116.5 (3)
C13—C12—H12	120.4	C6—C7—H7	121.7
C14—C13—C12	121.4 (2)	C8—C7—H7	121.7
C14—C13—H13	119.3	N3—C8—C7	132.2 (2)
C12—C13—H13	119.3	N3—C8—C9	106.2 (2)
C13—C14—C110^i^	120.9 (2)	C7—C8—C9	121.5 (2)
C13—C14—H14	119.6	N1—C9—C8	106.8 (2)
C110^i^—C14—H14	119.6	N1—C9—C4	131.4 (3)
C14^i^—C110—C110^i^	118.7 (3)	C8—C9—C4	121.7 (3)
C14^i^—C110—C11	123.1 (2)	F1A—C21—F2A	110.9 (8)
C110^i^—C110—C11	118.3 (3)	F3—C21—F2	112.5 (5)
C2—N1—C9	107.7 (2)	F1A—C21—F3A	109.7 (11)
C2—N1—H1	126.2	F2A—C21—F3A	99.4 (8)
C9—N1—H1	126.2	F3—C21—F1	106.2 (5)
C2—N3—C8	108.1 (2)	F2—C21—F1	103.2 (5)
C2—N3—H3	126.0	F3—C21—C2	113.7 (4)
C8—N3—H3	126.0	F2—C21—C2	110.7 (3)
N3—C2—N1	111.2 (2)	F1—C21—C2	109.8 (4)
N3—C2—C21	124.5 (2)		
			
O3—S1—C11—C12	−8.3 (2)	C6—C7—C8—N3	179.7 (3)
O2—S1—C11—C12	112.8 (2)	C6—C7—C8—C9	−0.4 (4)
O1—S1—C11—C12	−130.1 (2)	C2—N1—C9—C8	−0.2 (3)
O3—S1—C11—C110	174.97 (18)	C2—N1—C9—C4	−179.7 (3)
O2—S1—C11—C110	−63.9 (2)	N3—C8—C9—N1	−0.3 (3)
O1—S1—C11—C110	53.2 (2)	C7—C8—C9—N1	179.8 (2)
C110—C11—C12—C13	−1.5 (4)	N3—C8—C9—C4	179.2 (2)
S1—C11—C12—C13	−178.29 (19)	C7—C8—C9—C4	−0.7 (4)
C11—C12—C13—C14	1.4 (4)	C5—C4—C9—N1	−179.5 (3)
C12—C13—C14—C110^i^	−0.4 (4)	C5—C4—C9—C8	1.1 (4)
C12—C11—C110—C14^i^	−179.0 (2)	N3—C2—C21—F1A	176.3 (14)
S1—C11—C110—C14^i^	−2.3 (3)	N1—C2—C21—F1A	−2.1 (15)
C12—C11—C110—C110^i^	0.6 (4)	N3—C2—C21—F3	−25.8 (8)
S1—C11—C110—C110^i^	177.3 (2)	N1—C2—C21—F3	155.8 (7)
C8—N3—C2—N1	−0.9 (3)	N3—C2—C21—F2A	50.4 (10)
C8—N3—C2—C21	−179.5 (2)	N1—C2—C21—F2A	−128.0 (10)
C9—N1—C2—N3	0.7 (3)	N3—C2—C21—F2	101.9 (8)
C9—N1—C2—C21	179.3 (2)	N1—C2—C21—F2	−76.5 (8)
C9—C4—C5—C6	−0.5 (5)	N3—C2—C21—F3A	−58.3 (10)
C4—C5—C6—C7	−0.6 (5)	N1—C2—C21—F3A	123.3 (10)
C5—C6—C7—C8	1.0 (5)	N3—C2—C21—F1	−144.7 (5)
C2—N3—C8—C7	−179.3 (3)	N1—C2—C21—F1	36.9 (6)
C2—N3—C8—C9	0.7 (3)		

Symmetry codes: (i) −*x*+2, −*y*+1, −*z*.

### Hydrogen-bond geometry (Å, °)

**Table:**

*D*—H···*A*	*D*—H	H···*A*	*D*···*A*	*D*—H···*A*
N3—H3···O1	0.86	1.81	2.661 (3)	172
N1—H1···O2^ii^	0.86	1.84	2.650 (3)	155
C12—H12···O3^iii^	0.93	2.55	3.440 (3)	159

Symmetry codes: (ii) *x*−1, *y*, *z*; (iii) −*x*+2, −*y*+1, −*z*+1.
